# Fight Against the Mandatory COVID-19 Immunity Passport on Twitter: Natural Language Processing Study

**DOI:** 10.2196/49435

**Published:** 2023-11-23

**Authors:** Jessica S M Gable, Romy Sauvayre, Cédric Chauvière

**Affiliations:** 1 LAPSCO CNRS Université Clermont Auvergne Clermont-Ferrand France; 2 Polytech Clermont Clermont Auvergne INP Université Clermont Auvergne Aubiere France; 3 Laboratoire de Mathématiques Blaise Pascal (LMBP) CNRS UMR 6620 Université Clermont Auvergne Clermont-Ferrand France

**Keywords:** mandatory vaccination, public policy, public health measures, COVID-19, vaccine, social media analysis, Twitter, natural language processing, deep learning, social media, public health, vaccination, immunity, social distancing, neural network, effectiveness

## Abstract

**Background:**

To contain and curb the spread of COVID-19, the governments of countries around the world have used different strategies (lockdown, mandatory vaccination, immunity passports, voluntary social distancing, etc).

**Objective:**

This study aims to examine the reactions produced by the public announcement of a binding political decision presented by the president of the French Republic, Emmanuel Macron, on July 12, 2021, which imposed vaccination on caregivers and an immunity passport on all French people to access restaurants, cinemas, bars, and so forth.

**Methods:**

To measure these announcement reactions, 901,908 unique tweets posted on Twitter (Twitter Inc) between July 12 and August 11, 2021, were extracted. A neural network was constructed to examine the arguments of the tweets and to identify the types of arguments used by Twitter users.

**Results:**

This study shows that in the debate about mandatory vaccination and immunity passports, mostly “con” arguments (399,803/847,725, 47%; *χ*^2^_6_=952.8; *P*<.001) and “scientific” arguments (317,156/803,583, 39%; *χ*^2^_6_=5006.8; *P*<.001) were used.

**Conclusions:**

This study shows that during July and August 2021, social events permeating the public sphere and discussions about mandatory vaccination and immunity passports collided on Twitter. Moreover, a political decision based on scientific arguments led citizens to challenge it using pseudoscientific arguments contesting the effectiveness of vaccination and the validity of these political decisions.

## Introduction

During the COVID-19 pandemic, SARS-CoV-2 spread throughout the world and caused 652 million confirmed cases and more than 6.7 million deaths between January 6, 2020, and December 23, 2022 [1]. To contain and curb the spread of COVID-19, the governments of countries around the world used different strategies, such as lockdown, mandatory vaccination, immunity passports, voluntary social distancing, mask mandates, minimal room crowding, contact restrictions, and hygiene measures [2-7]. These obligations posed ethical questions [8] and generated a dynamic of change in people’s choices and behaviors regarding vaccination and various restrictive measures [9]. A lack of ambition in governmental health campaigns, and more specifically in vaccination programs, can reinforce vaccine refusal behaviors [10]. However, when carried out effectively, these public health measures can positively influence vaccination coverage [11-17]. In a counterintuitive dynamic, abandonment of coercive measures can lead to an increased acceptance of vaccination [6]. Moreover, the dynamics fluctuate under the influence of scientific or political speeches broadcast through mainstream media (newspapers), social media, or the internet [18,19].

This study aims to examine the reactions produced by the public announcement of a binding political decision presented by the president of the French Republic on July 12, 2021. In the early evening, Emmanuel Macron appeared on French television to announce two major constraints: (1) the mandatory use of an immunity passport to access restaurants, cinemas, theaters, museums, bars, swimming pools, long-distance trains, and so forth and (2) mandatory vaccination for caregivers with sanctions such as license revocation and nonpayment of salary. Five days later (July 17, 2021), anti–health measure protests began and mobilized more than 100,000 citizens. On July 25, the National Assembly adopted a law ratifying President Macron’s decisions. In this turbulent social and societal context, this study aims to use neural networks and natural language processing (NLP) to examine the reactions of users of the social network Twitter during the month following this July 12 announcement.

## Methods

### Data Collection

To measure the reactions produced by the French president’s measures, messages posted on Twitter, namely, tweets, between July 12 and August 11, 2021, were extracted with the following request: “vaccin lang: fr,” which included vaccines, vaccination, vaccinated, and so forth. This time span was chosen to allow proper measurement of the reactions following Macron's announcement on Twitter users, namely, tweeters. A previous study focusing on the analysis of the dissemination of messages during the COVID-19 pandemic [20] shows that the activity surrounding a tweet decreases very sharply, in 4 hours (for a retweet) or 3 days (for a quote), following its publication on Twitter. In addition, July 2021 was the month with the highest number of tweets on vaccination during the entire COVID-19 pandemic, with a growth rate of 142% over the previous month. Consequently, this 1-month duration is long enough to measure the chosen effect and short enough to avoid measuring other, parasitic effects. Our knowledge of the social context of the debate on the mandatory immunity passport confirms this methodological choice.

**Figure 1:**
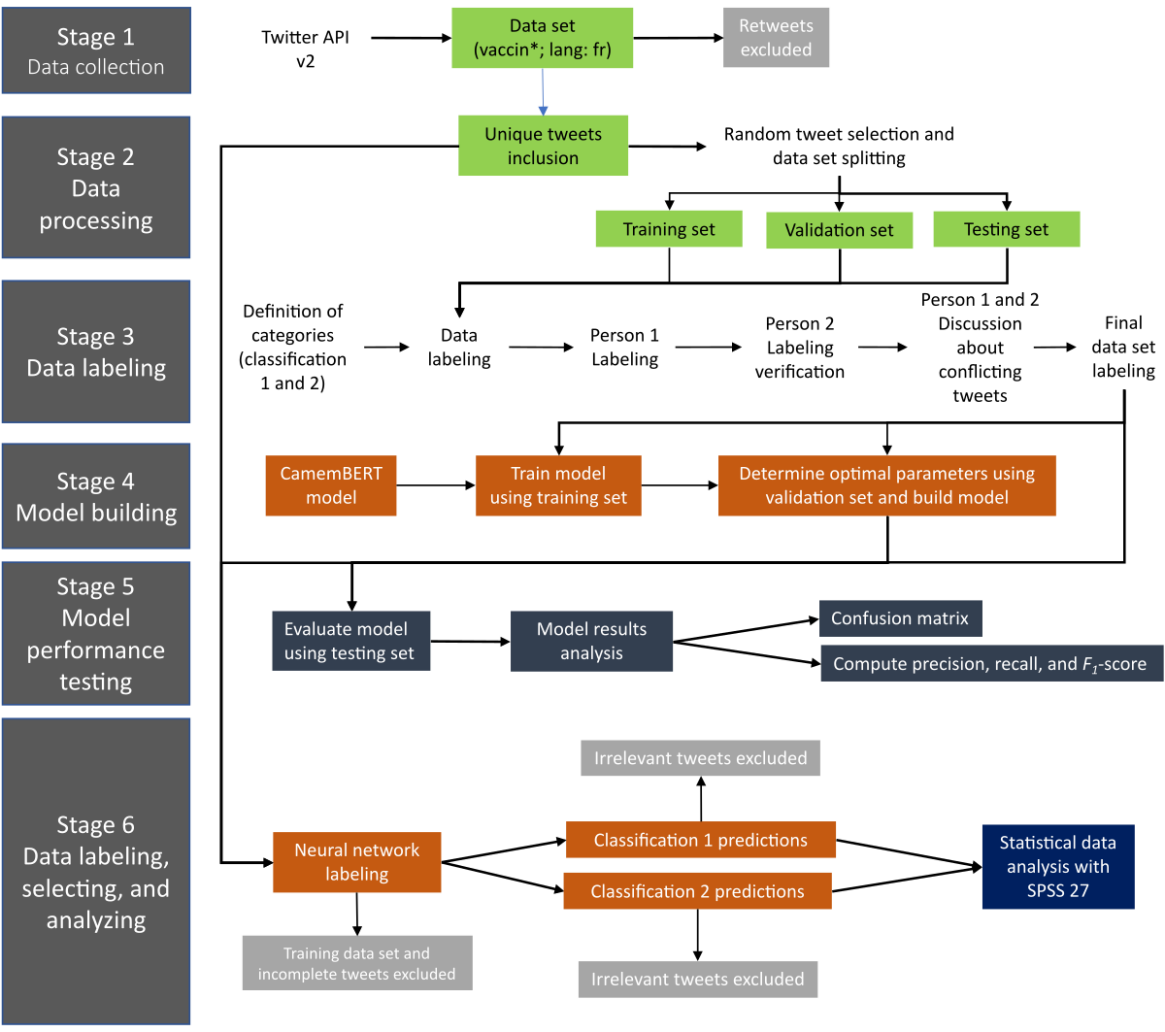
Flowchart of the study methodology.

After the tweets were downloaded, 1,782,176 were stored in a database. The data set contained 901,908 unique tweets published by more than 231,373 unique tweeters (see the complete flowchart in Figure 1). Unique tweets and retweets were identified by applying a Python (Python Software Foundation) script to the Twitter API. No duplicates were identified during the labeling phase of more than 1800 randomly selected tweets.

### Multiclass Classification

To examine the arguments of the tweets, two classifications were developed: (1) arguments for (pros) or against (cons) vaccination or health measures for sentiment analysis and (2) the types of argument used by the tweeters, such as scientific, political, or social. The criteria of the multiclass classifications are provided in Multimedia Appendix 1 with translated examples. In accordance with Twitter’s terms of use under the European General Data Protection Regulation, tweets cannot be shared [21].

### Neural Network and Accuracy

Over the past 10 years or so, neural networks have become versatile tools for solving a wide variety of problems, such as regression, classification, and reinforcement learning. The interested reader should refer to the introduction of *Deep Learning with Python* [22] for an up-to-date review. For this study, a pretrained French language neural network model called CamemBERT was used. It was released in 2020 and is considered one of the state-of-the-art French language models [23]. It relies heavily on transformers [24], which brought about nothing short of a revolution in the NLP field. The CamemBERT belongs to the Bidirectional Encoder Representations from Transformers (BERT) family of models, which are general multipurpose pretrained models that may be used for classification, question answering, translation, and so on. In this study, the CamemBERT model was fine-tuned to make it suitable for classification (see [25] for the comparison of the model performance). PyTorch (Meta AI) implementation of the large version of the CamemBERT model was downloaded from the Hugging Face [26]. When using deep learning models, the text has to be converted to numerical data. For that, we use the specific CamemBERT tokenizer from the Hugging Face. For training, validation, and testing, a total of 1851 unique tweets (randomly selected) were manually labeled by 2 people. As is customary when building a machine learning model, the data set was divided into 3 parts, and we used 1306 tweets for training (ie, fine-tuning the parameters of the model), 145 tweets to set the hyperparameters (essentially, the number of epochs), and 400 tweets to test the final model.

After fine-tuning CamemBERT, we obtained 59% (236/400) accuracy (*F*_1_-score 55.3%) for classification 1 and 67.6% (270/400) accuracy (*F*_1_-score 62.9%) for classification 2. Those results were based on the 400 tweets of the test data set. They were consistent with the results of other recently published studies (see Sauvayre et al [27] and Dupuy-Zini et al [28] for detailed references).

### Data Selection

Once the tweets were categorized by the model, all tweets had a label. Then, pursuant to the aim of this study, the labels most relevant to the 2 classifications, that is, the tweets “pro,” “con,” and “noncommittal” for classification 1 and “scientific,” “political,” and “social” for classification 2, were selected and analyzed with SPSS (version 27; IBM Corp) software (Multimedia Appendix 1).

### Ethical Considerations

In accordance with the European General Data Protection Regulation, this data collection has been registered with the Data Protection Officer of the French National Center for Scientific Research (treatment number 2-22120). In addition, all tweets have been anonymized. To ensure the anonymity of tweeters, translated examples of tweets have been provided with some adjustments.

## Results

### Overview

Most of the tweets (399,803/847,725, 47%; Table 1) were against political measures (immunity passport and mandatory vaccination) or expressed doubts about the effectiveness of the vaccine. In contrast, the arguments used by the tweeters (classification 2) were balanced between scientific (317,156/803,583, 39%), political (245,515/803,583, 31%), and social (240,912/803,583, 30%).

**Table 1:** Neural network prediction of classification 1 and classification 2.

Type of classification		Number of tweets, n (%)
**Classification 1 sentiment**		
	Noncommittal	180,288 (21)
	Pro	267,634 (32)
	Con	399,803 (47)
**Classification 2 arguments**		
	Scientific	317,156 (39)
	Political	245,515 (31)
	Social	240,912 (30)

### Sentiment and Argument Time Lines

**Figure 2:**
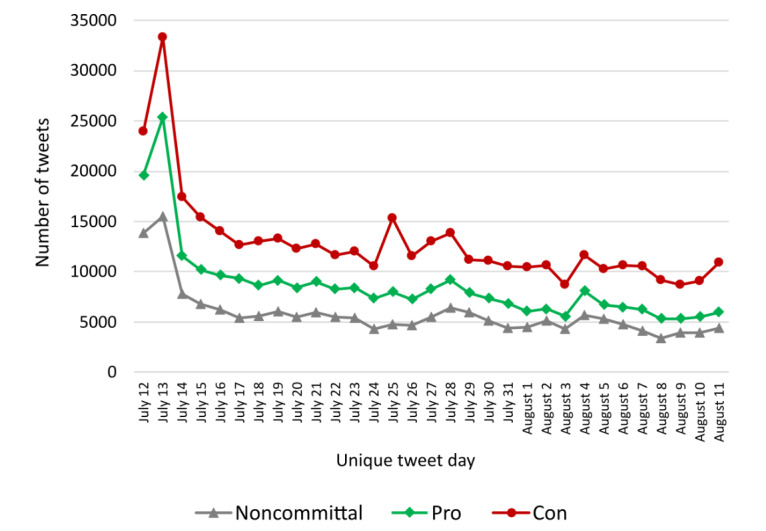
Time series of classification 1 sentiment (pro, con, and noncommittal) from July 12 to August 11, 2021, inclusive.

**Figure 3:**
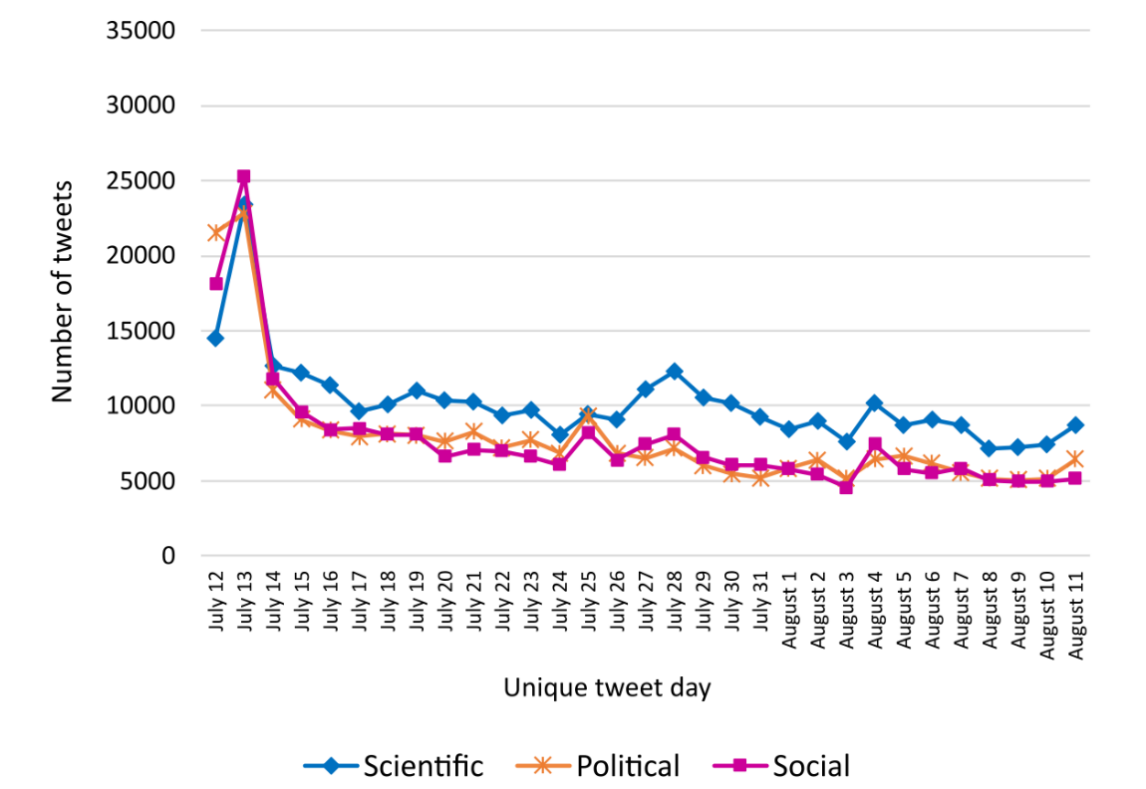
Time series of classification 2 (scientific, political, and social) from July 12 to August 11, 2021, inclusive.

The time series of classification 1 (Figures 2 and 3) were examined. The impact of the French president’s announcement was stronger on July 13, the day after his public declaration. Moreover, whatever the date, “con” tweets were more numerous than other types of tweets (“pro” or “noncommittal”). Indeed, there is a statistical link between classification 1 and the dates of the tweets grouped every 4 weeks (*χ*^2^_6_=952.8; *P*<.001).

Classification 2 focuses on the arguments of the tweets. We expected to see more political tweets following the announcement of a political decision. However, except for those posted on the first day, July 12, the tweets contained more scientific than political arguments. Indeed, there is a statistical link between classification 2 and the dates of the tweets grouped every 4 weeks (*χ*^2^_6_=5006.8; *P*<.001). These data show that the main argument used and spread on Twitter after the political health measure was scientific and not political.

**Figure 4:**
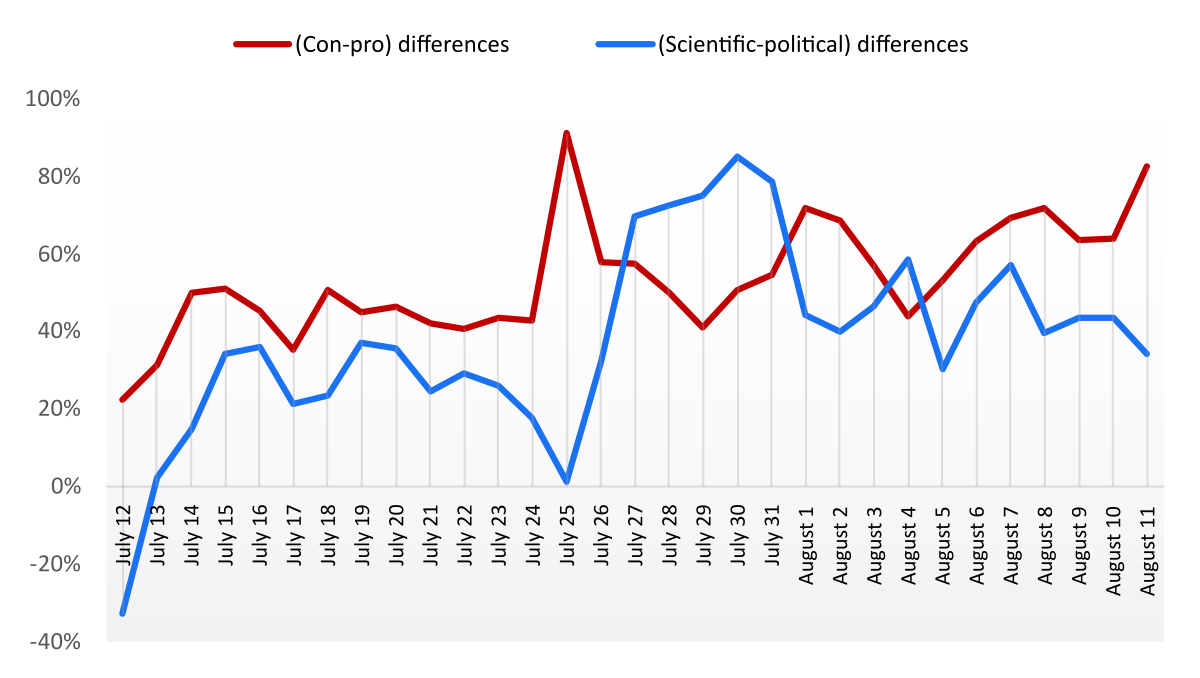
Time series of relative differences between con and pro (red line) and scientific and political (blue line) arguments.

In addition, the relative difference between the most frequent arguments (“con” and “scientific”) in each category and the most contested (“pro” and “political”) made it possible to identify a particular date, that is, July 25 (Figure 4). On July 25, the “con” tweets experienced a significant increase of 91% (15,313/8017; Multimedia Appendix 2). On the other hand, “scientific” tweets stood out from “political” tweets at the end of July (27 to 31), with a difference oscillating between 70% (11,122/6551) and 85% (10,149/5487; Multimedia Appendix 3).

## Discussion

### Principal Findings

The goal of this study is to examine the reactions following the public announcement of a binding political decision presented by the president of the French Republic, Emmanuel Macron, on July 12, 2021, and the arguments used by French-speaking users of Twitter. The main results show that published tweets were more likely to be negative than positive and more scientific than political.

### The Mandatory Effect

Vaccination obligations and sanctions had different levels of acceptance within different populations. Indeed, although half of Polish citizens accepted mandatory vaccinations, only one-quarter accepted the sanctions imposed by the Polish government [29]. However, in Finland, the more a person favored mandatory vaccination, the more they favored sanctions [30]. The inhabitants of South America (Colombians and Salvadorans) accepted mandatory vaccination more than Spaniards [31]. However, only 27.8% of Cypriots accepted mandatory vaccination [4] versus 73% of Germans [32]. The introduction of the immunity passport in France increased vaccination coverage against COVID-19 [16], but this increase did not mean acceptance of the health measures. This can be seen by examining the arguments that spread on Twitter during the month following President Macron’s announcement. Indeed, the examined tweets more often contained arguments against health measures and against mandatory vaccination (399,803/847,725, 47%), and they more often contained scientific arguments to justify their point of view (317,156/803,583, 39%). Disagreement with public policies, in particular the vaccine passports, was also found in English negative tweets examined between April 1, 2021, and August 1, 2022 [33].

One might expect tweets about vaccination to be more often negative because oppositional tweets would be more active. However, the literature is not consensual on this topic: studies analyzing tweets using NLP, similar to this study, discovered a majority of positive tweets [34], but others discovered a majority of negative tweets [33,35]. In our study, the discourse of French speakers collected on Twitter showed how strongly the French were divided about health measures in the summer of 2021.

### Dynamics of the Debate

One might expect a political decision to engender more political arguments. However, President Macron’s political decision quickly became a topic of scientific debate. Users in support of health measures presented mainstream scientific arguments, while users opposing such measures presented distorted and contested versions of these arguments, corresponding to the wave of disinformation identified on social media [36]. It therefore seems that the legitimacy of a public health decision fueled by a scientific discourse leads tweeters to attack the scientific arguments on which the decision is based rather than the political decision itself.

### Mandatory Vaccination for Caregivers

The collected data showed that a significant increase in the opposition rate occurred on 1 date in particular: July 25, 2021, the date on which the French government enacted a law requiring caregivers to be vaccinated and including penalties. While the obligation for caregivers (including clinicians) to be vaccinated was presented as an ethical necessity to protect their patients [37], this question divided the French. On Twitter, French-speaking tweeters expressed their disagreement with the deprivation of a fundamental right, namely, individual freedom. The debate then focused on opinions in opposition to this measure, mobilizing both scientific and political arguments.

### Science Divides Twitter Users

The dynamics of the debates at the end of July 2021 took a more scientific turn because the main justification for the mandatory caregiver vaccination lay in a scientific argument. This argument was that people who are vaccinated spread less virus than people who are not vaccinated, in short, that the vaccine limits the spread of the virus. These arguments were then strongly debated on Twitter at the end of July. On July 30, an article published in a blog on the Mediapart newspaper website claimed that the COVID-19 vaccine was dangerous [38]. The article was quickly withdrawn, but the idea was relayed on Twitter. Finally, on July 31, 2021, the New York Times [39] relayed a report from the Centers for Disease Control and Prevention (CDC) claiming that people who are vaccinated transmitted SARS-CoV-2 more than others. The article was then posted on the CDC website [40] and fueled the heated “pros” and “cons” debates on Twitter. Finally, Macron’s debate, occurring in July and August 2021, shows that pseudoscience arguments were used to contest political measures. The consequences of pseudoscience dissemination on social media are to possibly lead more individuals to avoid the health care system and might increase their medical care delay and the mortality risk of these social media’s skeptical patients.

### Limitations

This study has several limitations. First, the data were collected only from Twitter, which is a specific social media platform. Even if the message reflects the debate occurring in the “real word,” the result cannot be generalized to other social media. Second, the study focused on tweets containing the word “vaccine” and its derivatives without further selections. Third, the neural network methodology used needs to be improved to analyze very short texts such as tweets. Fourth, the data were collected on a specific time span (July and August), which reflects a specific moment of the debate about mandatory vaccines. Further research needs to be conducted to generalize the obtained results about the consequences of a political measure justified by scientific arguments.

### Conclusions

This study shows that during July and August 2021, social events permeating the public sphere and discussions about mandatory vaccination and immunity passports collided on Twitter. In France, binding political decisions to contain it was presented to the French on July 12. The antisanitary pass demonstrations and the spreading of pseudoscientific theories contesting the effectiveness of vaccination occurred during the rebound of the COVID-19 pandemic. In this context, binding political decisions caused a resurgence of messages on Twitter, mobilizing arguments against vaccination and the health record and scientific arguments challenging the validity of these decisions.

## Appendix

Multimedia Appendix 1 Classification criteria and definitions.

Multimedia Appendix 2 Number of tweets as a function of the classification 1.

Multimedia Appendix 3 Number of tweets as a function of the classification 2.

## Abbreviations

BERT: Bidirectional Encoder Representations from Transformers
CDC: Centers for Disease Control and Prevention
NLP: natural language processing

## Data Availability

The data sets analyzed during this study are available from the corresponding author on reasonable request. The data sets generated during this study are not publicly available due to the Twitter’s terms of use under the European General Data Protection Regulation, tweets cannot be shared [21]. In addition, the full codes used to create the neural network are available on GitHub [41].
