# Supplementary material for: Fight Against the Mandatory COVID-19 Immunity Passport on Twitter: Natural Language Processing Study
Source: J Med Internet Res. 2023 Nov 23;25:e49435. doi: 10.2196/49435 (PMC10669926; doi:10.2196/49435)
Supplement: Multimedia Appendix 1 [file jmir_v25i1e49435_app1.pdf]

Multimedia Appendix 1

Classification Criteria and Definitions

Type of classification	Definition	Translated examples (French to English)
**Classification 1**		
Unclassifiable	Unclassifiable or irrelevant for the topic “vaccination” or “sanitary measures”.	The Emmanuel Macron effect.
Noncommittal	Neutral or without explicit opinion on vaccination and/or on the sanitary pass.	I have to ask my doctor for the vaccine.
Pro	Arguments in favor and/or on the sanitary pass<br>Arguments in favor of the benefits of the COVID-19 vaccine and/or on the sanitary pass (efficiency, safety, relevance).	Personally, I am vaccinated so nothing to fear, on the other hand, good luck to all the anti-vaccine, you will not have the choice now??
Con	Arguments against vaccination or doubts about the effectiveness of the COVID-19 vaccine, fear of side effects and refusal of the sanitary pass.	I am against the vaccine I am not afraid of the virus but I am afraid of the vaccine.
**Classification 2**		
Unclassifiable	Irrelevant or unclassifiable.	A vaccine.
Scientific	Scientific or pseudoscientific content that using true beliefs or false information.	The vaccine is 95% efficient, a little less in fragile people. The risk is not zero, but a vaccinated person has much less chance of transmitting the virus.

Type of classification	Definition	Translated examples (French to English)
Political	Comments on a legal or political decisions about vaccination or sanitary measures.	Basically the vaccine is mandatory, shameful LMAO.
Social	Comments, debates or gives an opinion on the report to other members of society.	"Pro vaccine" you have to also understand that there is people who does not want to be vaccinated.
Vaccination status	Explicit tweet about the vaccination status of the tweeter's users.<br>Comments on the symptoms experienced after COVID-19 vaccine injection.<br>Explicit refusing a COVID-19 vaccine.	Ex.1: I am very glad to have already done my 2 doses of the vaccine, fudge.<br>Ex.2: I don't want to get vaccinated. Why? Well, you know, we don't know what's in this vaccine, it can be dangerous.
