# Supplementary material for: Fight Against the Mandatory COVID-19 Immunity Passport on Twitter: Natural Language Processing Study
Source: J Med Internet Res. 2023 Nov 23;25:e49435. doi: 10.2196/49435 (PMC10669926; doi:10.2196/49435)
Supplement: Multimedia Appendix 2 [file jmir_v25i1e49435_app2.pdf]

Multimedia Appendix 2

Number of tweets as a function of the classification 1

Days	Non committal	Pros	Cons	(Cons - Pros) Differences
July 12	13,827	19,602	23,972	22%
July,13	15,524	25,363	33,327	31%
July 14	7,754	11,604	17,406	50%
July 15	6,747	10,208	15,418	51%
July 16	6,228	9,654	14,016	45%
July 17	5,416	9,348	12,664	35%
July 18	5,591	8,651	13,042	51%
July 19	6,030	9,156	13,277	45%
July 20	5,504	8,394	12,280	46%
July 21	5,925	9,003	12,783	42%
July 22	5,525	8,298	11,687	41%
July 23	5,372	8,404	12,052	43%
July 24	4,348	7,348	10,508	43%
July 25	4,790	8,017	15,313	91%
July 26	4,671	7,287	11,516	58%
July 27	5,507	8,265	13,031	58%
July 28	6,411	9,205	13,813	50%
July 29	5,940	7,933	11,176	41%
July 30	5,114	7,348	11,069	51%
July 31	4,375	6,837	10,578	55%
August 1	4,498	6,074	10,446	72%
August 2	5,145	6,308	10,634	69%
August 3	4,332	5,561	8,743	57%
August 4	5,723	8,077	11,629	44%
August 5	5,348	6,735	10,320	53%
August 6	4,764	6,499	10,613	63%
August 7	4,135	6,251	10,584	69%
August 8	3,430	5,349	9,185	72%
August 9	3,964	5,349	8,757	64%
August 10	3,981	5,540	9,075	64%
August 11	4,369	5,966	10,889	83%
