# Supplementary material for: Fight Against the Mandatory COVID-19 Immunity Passport on Twitter: Natural Language Processing Study
Source: J Med Internet Res. 2023 Nov 23;25:e49435. doi: 10.2196/49435 (PMC10669926; doi:10.2196/49435)
Supplement: Multimedia Appendix 3 [file jmir_v25i1e49435_app3.pdf]

Multimedia Appendix 3

Number of tweets as a function of the classification 2

Days	Scientific	Political	Social	(Scientific - Political) Difference
July 12	14,502	21,551	18,105	-33%
July 13	23,367	22,802	25,309	2%
July 14	12,677	11,017	11,789	15%
July 15	12,236	9,112	9,603	34%
July 16	11,392	8,373	8,428	36%
July 17	9,657	7,952	8,510	21%
July 18	10,052	8,136	8,081	24%
July 19	11,048	8,053	8,110	37%
July 20	10,349	7,634	6,615	36%
July 21	10,318	8,276	7,089	25%
July 22	9,364	7,244	7,011	29%
July 23	9,753	7,739	6,621	26%
July 24	8,075	6,854	6,080	18%
July 25	9,457	9,325	8,251	1%
July 26	9,091	6,861	6,415	33%
July 27	11,122	6,551	7,444	70%
July 28	12,339	7,157	8,117	72%
July 29	10,568	6,036	6,581	75%
July 30	10,149	5,487	6,074	85%
July 31	9,277	5,197	6,093	79%
August 1	8,450	5,860	5,811	44%
August 2	8,959	6,405	5,434	40%
August 3	7,579	5,175	4,552	46%
August 4	10,219	6,445	7,440	59%
August 5	8,735	6,695	5,786	30%
August 6	9,115	6,176	5,525	48%
August 7	8,740	5,565	5,853	57%
August 8	7,191	5,148	5,078	40%
August 9	7,263	5,059	4,976	44%
August 10	7,435	5,175	4,968	44%
August 11	8,677	6,455	5,163	34%
